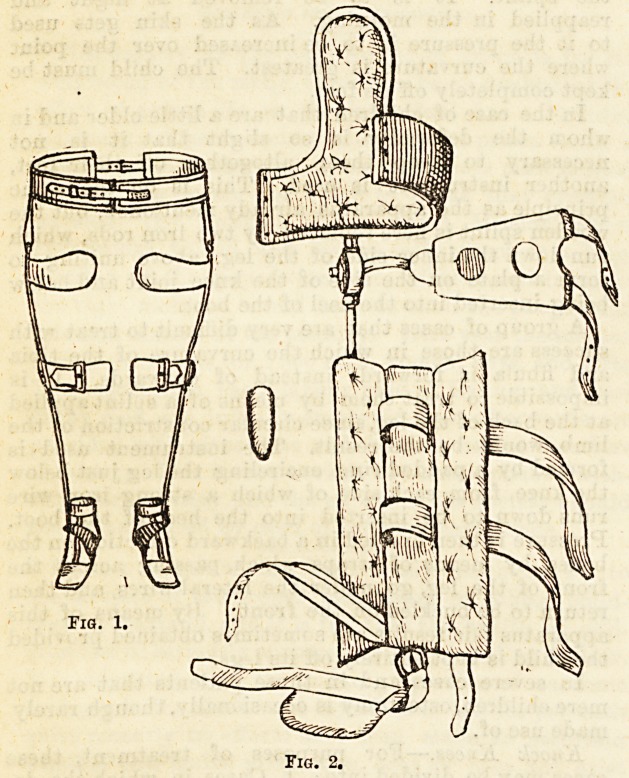# The Treatment of Flat Foot, Bow Legs, and Knock Knee

**Published:** 1893-04-08

**Authors:** 


### THE HOSPITAL CLINIC.

[*The Editor will be glad to receive offers of co-operation and contributions from members of the profession All letters should be addressed to* THE EDITOR, THE LODGE, PORCHESTER SQUARE, LONDON, W.]

### THE ROYAL ORTHOPÆDIC HOSPITAL.

### THE TREATMENT OF FLAT FOOT, BOW LEGS, AND KNOCK KNEE.

*Flat Foot.*—As far as treatment is concerned, cases of flat foot are regarded at the Royal Orthopædic Hospital as being of two classes. The first contains those cases in which there is no rigidity and the arch can be restored by pressure. In the second are those cases in which there is rigidity and the arch cannot be restored by pressure.

Cases of the first sort are treated by means of a special boot, which contains a sole plate. The boot is large, so as to allow plenty of room for the foot. Its inner edge is quite straight, and the heel is broad, low, and carried well forwards. The sole plate consists of a steel plate shaped like the under surface of a natural foot. It is turned up on the inner side where the arch of the foot ought to be, and here is padded with several layers of indiarubber. When worn in the boot it is kept in position by the weight of the body on it, and the direct pressure of the padded part supports the arch.

In addition, the patient is advised to rest as much as possible, and is given a simple lin iment wherewith to rub the foot and the muscles of the calf of the leg, which in some of these cases are wasted.

With regard to the second class of cases—that is to say, those in which there is rigidity and the arch cannot be restored by pressure, a much more elaborate treatment is required. To begin with, the foot is kept completely at rest for a few days. In some cases the rigidity passes off, and the case can be treated like those already described. In most cases, however, although the pain may cease the rigidity remains. If the tendons of the peronei muscles are felt to be tight as they pass round the outer ankle they are divided, and the foot is kept for a few days at rest on a splint. The splint is then removed, the foot well rubbed with liniment, and pressure made in a way tending to replace the sunken arch. This is done every day for a few days, and then, when the wound has healed, the rubbing with liniment is frequently repeated, and the joints of the foot are thoroughly wrenched, if necessary, under an anæsthetic. After a short time it is generally possible to replace the arch, and the patient is ordered the apparatus shown. The boot and sole plate are just the same as in the previous cases, but, in addition, there is a strong iron on the outside of the leg which runs from the heel of the boot to just below the knee, and the foot is braced to this by a strong T-shaped strap. By this instrument the pressure is removed from the inner side of the foot at the same time that the arch is directly supported. The manipulation of the foot and the rubbing with liniment are still continued for some time.

*Bow Legs.*—In these cases there is generally found some error of diet. If so, it is corrected, and then attention is given to the treatment of the deformity. If the tibia and fibula are simply bent outwards, a broad well-padded splint is provided, long enough to reach from just below the sole of the foot to about an inch above the knee. The splint is applied with straps to the inner side of the leg. In order that this treatment may be successful it is necessary that attention be given to the proper application of the splint. It is to be removed at night and reapplied in the morning. As the skin gets used to it the pressure is to be increased over the point where the curvature is greatest. The child must be kept completely off its feet.

In the case of children that are a little older and in whom the deformity is so slight that it is not necessary to keep them altogether off their feet, another instrument is used. This is on the same principle as the apparatus already mentioned, but the wooden splint is here replaced by two iron rods, which run down the inner side of the leg, above uniting to form a plate on the side of the knee joint and below being inserted into the heel of the boot.

A group of cases that are very difficult to treat with success are those in which the curvature of the tibia and fibula is forwards instead of outwards. It is impossible to treat them by means of a splint applied at the back of the leg, since circular constriction of the limb would be the result. The instrument used is formed by a padded ring encircling the leg just below the knee, from each side of which a strong iron wire runs down to be inserted into the heel of the boot. Pressure is then exerted in a backward direction on the bones by means of straps which, passing across the front of the leg, go under the lateral wires, and then return to be buckled on the front. By means of this apparatus fair results are sometimes obtained provided the child is kept entirely off its legs.

In severe cases and in those patients that are not mere children, osteotomy is occasionally, though rarely made use of.

*Knock Knees.*—For purposes of treatment, these cases may be divided into: 1. Cases in which the deformity is evident when the patient is walking, but in which it is possible to make the knees and the inner ankles touch one another when the patient is lying down. 2. Cases in which, when the patient is lying down and the knees are in apposition, it is impossible to bring the internal malleoli nearer together than two to three inches. 3. Cases in which the malleoli under the same circumstances are six, seven, or more inches apart.

In the first class there is often flat foot, and the genu valgum is sometimes the result of the increased strain thus thrown on the lateral ligaments of the knee. These cases are treated by means of a sole plate as described in the paragraph on the treatment of flat foot; and, if necessary, outside irons are ordered, which commence at the heel of the boot and run up the outside of the leg, to end in a band halfway between the knee and the hip. A leather strap at the knee draws the joint outwards towards the iron and so supports it.

In the second class, if the patient is young, it is usually treated by the use of wooden splints, which act very satisfactorily. The splints are long enough to reach from the waist to the sole of the foot. The upper end of each is perforated, and a strap is passed through the perforation. This band is carried across the back of the pelvis and sacrum and is attached to another coming from the opposite splint. The splints are in this way held back, and so cannot slip forwards. This is most important, as otherwise the splint slips forward, the leg becomes rotated, and all traction is removed from the knee. The splint is attached to the leg by broad straps and buckles, which surround it from the ankle to a little above the knee, and continually exert traction outwards on the joint. The splint is only worn during the day, and of course the child is kept off his feet as much as possible. In the case of older patients boots and irons are made use of. They only differ from those described under the previous heading in that they are generally carried up to a band round the waist, and that at the knee, instead of a hinge joint there is a mechanism worked by a screw whereby the amount of outward traction can be increased or diminished at will.

Fig. 1.

Fig. 2.

In the more severe cases in which the malleoli are seven, eight, or more inches apart the tendon of the biceps is generally divided. It is usually done without an anæsthetic, as it is thought there is thus less risk of injuring the external popliteal nerve. The patient is kept in bed, and the trough instrument shown in fig. 2 is applied. This instrument was suggested by Mr. Henry Baker. It consists essentially of a strong iron running down the outside of the leg. This is interrupted opposite the knee by a screw joint, capable of making traction in an outward direction on a strong strap that surrounds the joint. Above it takes purchase by means of a plate applied over the great trochanter. This is prevented from slipping forwards by a metal band passing behind the thigh. Below, the iron is attached to a sort of trough, which has a hinged wing on the inner side, and lower down a sole plate to support the foot at right angles with the leg. The sole plate has a strap fixed to its inner side, by means of which the foot can be turned inwards, and thus the ankle and leg prevented from rotating out. By means of this instrument, while the knee is kept extended and the leg prevented from rotating, lateral traction in an outward direction can be exerted on the knee joint with sufficient force to gradually overcome the deformity. This instrument is removed every day and the limb dusted with powder. It is then readjusted, and the traction on the knee is increased as far as is advisable.

The deformity having been corrected, the patient is kept at rest for some time in order to give the structures time to adjust themselves to the new conditions, and then the patient is allowed gradually to move about, the knee being supported by the instrument above described.

This process, if efficiently carried out, is extremely successful in the case of a child, its only drawbacks being that it is very tedious, and that the required instruments are rather expensive.

Osteotomy is sometimes, though very rarely, performed at the Royal Orthopædic Hospital for the cure of knock knees.

*Lateral Curvature of the Spine.*—This is treated by means of a spinal support. The instrument used is made of steel, and is at once both light and powerful. Pressure is made by means of plates on the projecting parts, and as the curve changes the plate is again brought to act on it by means of cog wheels. Recumbency is recommended as much as possible in all these cases, and various extension exercises are used; indeed, the lighter forms of the disease are treated entirely by their means. In addition, massage is applied to the muscles of the spine, with a view to improving their condition, and the general health, if in any way affected, is carefully attended to.

## Figures and Tables

**Figure f1:**
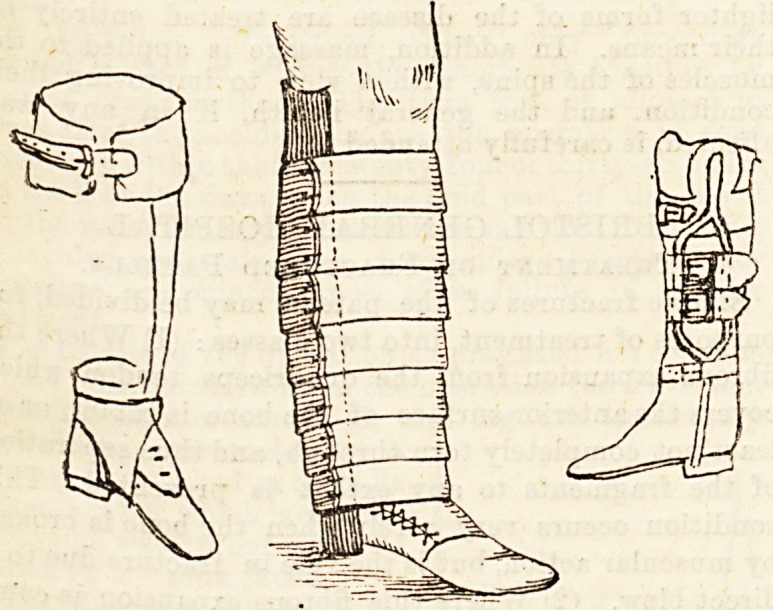


**Fig. 1. Fig. 2. f2:**